# Expression quantitative trait loci infer the regulation of isoflavone accumulation in soybean (*Glycine max* L. Merr.) seed

**DOI:** 10.1186/1471-2164-15-680

**Published:** 2014-08-13

**Authors:** Yan Wang, Yingpeng Han, Weili Teng, Xue Zhao, Yongguang Li, Lin Wu, Dongmei Li, Wenbin Li

**Affiliations:** Key Laboratory of Soybean Biology in Chinese Ministry of Education (key Laboratory of Soybean Biology and Breeding/Genetics of Chinese Agriculture Ministry), Northeast Agricultural University, Harbin, 150030 China

**Keywords:** Soybean, eQTL, Isoflavone, pQTL, Candidate genes

## Abstract

**Background:**

Mapping expression quantitative trait loci (eQTL) of targeted genes represents a powerful and widely adopted approach to identify putative regulatory variants. Linking regulation differences to specific genes might assist in the identification of networks and interactions. The objective of this study is to identify eQTL underlying expression of four gene families encoding isoflavone synthetic enzymes involved in the phenylpropanoid pathway, which are phenylalanine ammonia-lyase (PAL; EC 4.3.1.5), chalcone synthase (CHS; EC 2.3.1.74), 2-hydroxyisoflavanone synthase (IFS; EC1.14.13.136) and flavanone 3-hydroxylase (F3H; EC 1.14.11.9). A population of 130 recombinant inbred lines (F_5:11_), derived from a cross between soybean cultivar ‘Zhongdou 27’ (high isoflavone) and ‘Jiunong 20’ (low isoflavone), and a total of 194 simple sequence repeat (SSR) markers were used in this study. Overlapped loci of eQTLs and phenotypic QTLs (pQTLs) were analyzed to identify the potential candidate genes underlying the accumulation of isoflavone in soybean seed.

**Results:**

Thirty three eQTLs (thirteen cis-eQTLs and twenty trans-eQTLs) underlying the transcript abundance of the four gene families were identified on fifteen chromosomes. The eQTLs between Satt278-Sat_134, Sat_134-Sct_010 and Satt149-Sat_234 underlie the expression of both *IFS* and *CHS* genes. Five eQTL intervals were overlapped with pQTLs. A total of eleven candidate genes within the overlapped eQTL and pQTL were identified.

**Conclusions:**

These results will be useful for the development of marker-assisted selection to breed soybean cultivars with high or low isoflavone contents and for map-based cloning of new isoflavone related genes.

## Background

Soy food has been taken as a functional food because it contains many health beneficial molecules such as isoflavones [1]. Studies on human nutrition have shown that soybean isoflavones play an important role in preventing a number of chronic diseases [2, 3]. Equally, isoflavones are critical factors in defending soybean crops against pests [4, 5], in promoting nodulation by rhizobia [6], and in changing or adjusting the microorganisms around plant roots [7]. The major bioactive components of soybean isoflavones in human nutrition are daidzein (DZ), genistein (GT) and glycitein (GC). Isoflavone contents in soybean seed are inherited as complex quantitative traits [8–11]. Since soy seed isoflavones are regulated by multiple genetic factors, their concentrations in seed are highly variable [1, 12–14]. Over fifty QTLs underlying individual and/or total soybean isoflavone content have been reported [8, 15–23]. However, only 12 of these QTLs were in genomic regions encoding isoflavone synthesis enzymes.

A group of enzymes in the phenylpropanoid pathway lead to the biosynthesis of DZ, GT and GC [11]. Phenylalanine ammonia lyase (PAL; EC 4.3.1.5), chalcone synthase (CHS; EC 2.3.1.74) and flavanone 3-hydroxylase (F3H; EC 1.14.11.9) [24] are the first three enzymes that convert the amino acid phenylalanine into p-Coumaroyl-CoA in this pathway [11]. In the isoflavonoid biosynthetic pathway [25], the co-catalytic action of *CHS* [26, 27] with chalcone reductase (CHR; EC 2.3.1.170) [28] produces isoliquiritigenin and naringenin chalcone, which are isomers of the central isoflavanone intermediates naringenin and liquiritigenin, respectively. Isoliquiritigenin and naringenin chalcone are respectively converted into liquiritigenin and naringenin by chalcone isomerase (CHI; EC 5.5.1.6) [29]. These two products are the precursors of DZ and GT, which are formed after the catalysis of the precursors by the key enzyme 2-hydroxyisoflavanone synthase (IFS; EC 1.14.13.136) [30, 31]. The enzyme F3H, that competes with IFS in utilizing naringenin, catalyzes the conversion of flavanones to dihydroflavonols, which are intermediates in the biosynthesis of flavonols, anthocyanidins, catechins and proanthocyanidins [32, 33]. For the synthesis of GC, isoliquiritigenin is likely a precursor to form GC after several biochemical steps, which are not entirely known yet [34]. However, seed isoflavone concentrations in soybean can be regulated by metabolic engineering of the complex phenylpropanoid biosynthetic pathways [35].

Regulating transcript abundance is an effective approach to improve phenotypes [36]. The integrated analysis of genotype and transcript abundance data for association with complex traits can be used to identify novel genetic pathways involved in complex traits. ‘Expression QTL’ (eQTL), first defined by Jansen and Nap [37], could identify the genetic determinants of transcript abundances and is widely used for investigating gene regulation pathways. This approach treats transcript abundance of individual genes as quantitative traits in a segregating population. The eQTL map information enables genetic regulatory networks to be modeled that can provide a better understanding of the underlying phenotypic variation. It has been successfully applied in humans [38–40], plants [41–44], yeasts [45, 46], worms [47], flies [48], mice [49, 50], pigs [51] and rats [52] populations. These studies showed that transcript abundance was highly heritable and could be linked to either a local locus (cis-eQTL) or a distant locus (trans-eQTL). Cis-eQTL is mapped to the same genomic location like an expressed gene (within 5 Mb), and trans-eQTL is mapped to a different genomic location from an expressed gene (>5 Mb or on different chromosomes) [40, 53]. In general, cis-eQTL tends to produce stronger statistical associations than does by trans-eQTL [54]. This phenomenon is regarded as evidence of greater biological plausibility for the existence of true functional cis-eQTL [55]. Trans-eQTL could occur individually at a single genomic locus or could occur collectively as part of eQTL trans-bands [55]. This genomics approach has been employed to identify eQTL related genes in soybean [36, 56–58]. To date, no information concerning eQTLs underlying soybean isoflavone synthetic enzyme genes is available.

It has been proved that many enzymes in the phenylpropanoid pathway underlie QTLs that determine the accumulation of isoflavone contents in soybean seeds [11]. Meanwhile, the modification of enzyme encoded genes that are involved in phenylpropanoid pathway could promote the biosynthesis of isoflavone [31, 35]. In this study, *PAL*, *CHS*, *IFS* and *F3H* in the phenylpropanoid pathway were selected as the target genes (TGs) to analyze isoflavone-relative eQTL. Potential candidate genes underlying the accumulation of isoflavone contents in soybean seed were also evaluated. In addition, overlapped loci both for eQTL and phenotypic QTL (pQTL) were identified.

## Results

### Total and individual isoflavone contents, target gene transcript abundance and correlation analysis

Transcript abundances of target genes (TGs) between parents from R3 to R8 developmental stages were compared. Total and individual isoflavone contents and transcript abundances of TGs at R6 stage of soybean development were measured in the F_5:11_ population. The results showed that significant differences among the transcript abundances of TGs between the two parents existed at the R6 stage. The phenotypic variation of individual and total isoflavones showed a continuous distribution (Table 1).

**Table 1:** **Total and individual isoflavone content of the RIL populations and parents**

Traits^a^	Mean^b^	SD^b^	Min^b^	Max^b^	Zhongdou 27^c^	Jiunong 20^c^	Skewness	Kurtosis
DZ	9.61	3.04	4.36	15.88	8.92 ± 2.97	4.79 ± 1.12	−0.040	−0.765
GC	0.41	0.32	0.29	2.64	0.36 ± 0.16	0.42 ± 0.23	0.138	0.825
GT	4.38	2.55	0.77	9.51	4.22 ± 2.75	2.81 ± 1.01	0.480	−0.860
TI	14.40	5.21	5.70	25.11	13.50 ± 5.21	6.81 ± 2.27	0.187	−1.061
*PAL* expression(∆∆CT)	3.926	7.388	0.009	37.570	0.252	0	0.590	0.846
*CHS* expression(∆∆CT)	0.013	0.013	0.0002	0.051	0.328	0	1.203	0.616
*IFS* expression(∆∆CT)	0.896	1.334	0.002	5.199	0.707	0	0.954	1.700
*F3H* expression(∆∆CT)	4.798	3.481	0.013	10.550	10.550	−16.047	0.156	−1.340

^a^DZ, Daidzein; GC, Glycitein; GT, Genistein; TI, Total isoflavone content.

^b^μg/100 g(DZ, GC, GT, TI).

^c^Mean ± SD.

GT showed a high positive correlation coefficient with DZ (r = 0.762, P < 0.01; Table 2). The transcript abundance of *PAL* was positively correlated with both GT and TI, but exhibited no significant correlation with DZ and GC. The transcript abundance of *CHS* was positive correlated with DZ, GT and TI, but negatively associated with GC amount. The transcript abundance of *IFS* displayed a positive correlation with DZ, but showed no correlation with other isoflavone components. The transcript abundance of *F3H* showed significantly negative correlation with individual and total isoflavone contents.

**Table 2:** **Correlations among individual and total isoflavone contents, as well as the transcript abundances of the four TGs in the RIL populations**

Traits	DZ	GC	GT	TI	***PAL***expression	***CHS***expression	***IFS***expression
GC	0.249*						
GT	0.762**	0.294*					
TI	0.943**	0.363*	0.928**				
*PAL* expression	−0.094	0.092	0.269*	0.304*			
*CHS* expression	0.223*	−0.191*	0.201*	0.230*	0.063		
*IFS* expression	0.327*	−0.032	0.169	0.140	−0.022	0.022	
*F3H* expression	−0.248*	−0.248*	−0.276*	−0.273*	0.105	0.108	−0.001

P values were as follows: *P < 0.05, **P < 0.01.

### Identification of genomic region for target genes

Through BLAST searches (http://www.phytozome.net/soybean), the *PAL* has six homologous regions (E ≤ 0), which are located on Gm10 (LG O, *PAL1/ PAL2*), Gm13 (LG F, *PAL1*), Gm03 (LG N, *PAL1*), Gm19 (LG L, *PAL1*), Gm20 (LG I) and Gm02 (LG D1b, *PAL1*). Homologous regions encoding *CHS* (E-value ≤ 1.0E-05) are located on Gm11 (LG B1, *CHS8*), Gm01 (LG D1a, *CHS6/CHS7*), Gm08 (LG A2, *CHS1/CHS2/CHS3/CHS4/CHS5/CHS9*), Gm05 (LG A1, *CHS2*), Gm02 (LG D1b), Gm09 (LG K, *CHS6*), Gm19 (LG L) and Gm13 (LG F). Genes that encode *F3H* are located on Gm02 (LG D1b, *F3H1/F3H2*), Gm16 (LG J), Gm01 (LG D1a), Gm11 (LG B1), Gm18 (LG G) and Gm19 (LG L). Genes encoding *IFS* are located on Gm07 (LG M *IFS1*), Gm13 (LG F, *IFS2*), Gm10 (LG O), Gm03 (LG N), Gm12 (LG H), Gm19 (LG L), Gm17 (LG D2) and Gm11 (LG B1). Genes encoding *IFS* have the function of P450 cytochromes [27] and might have additional functional homologs.

### eQTL analysis for four TGs

**Figure 1:**
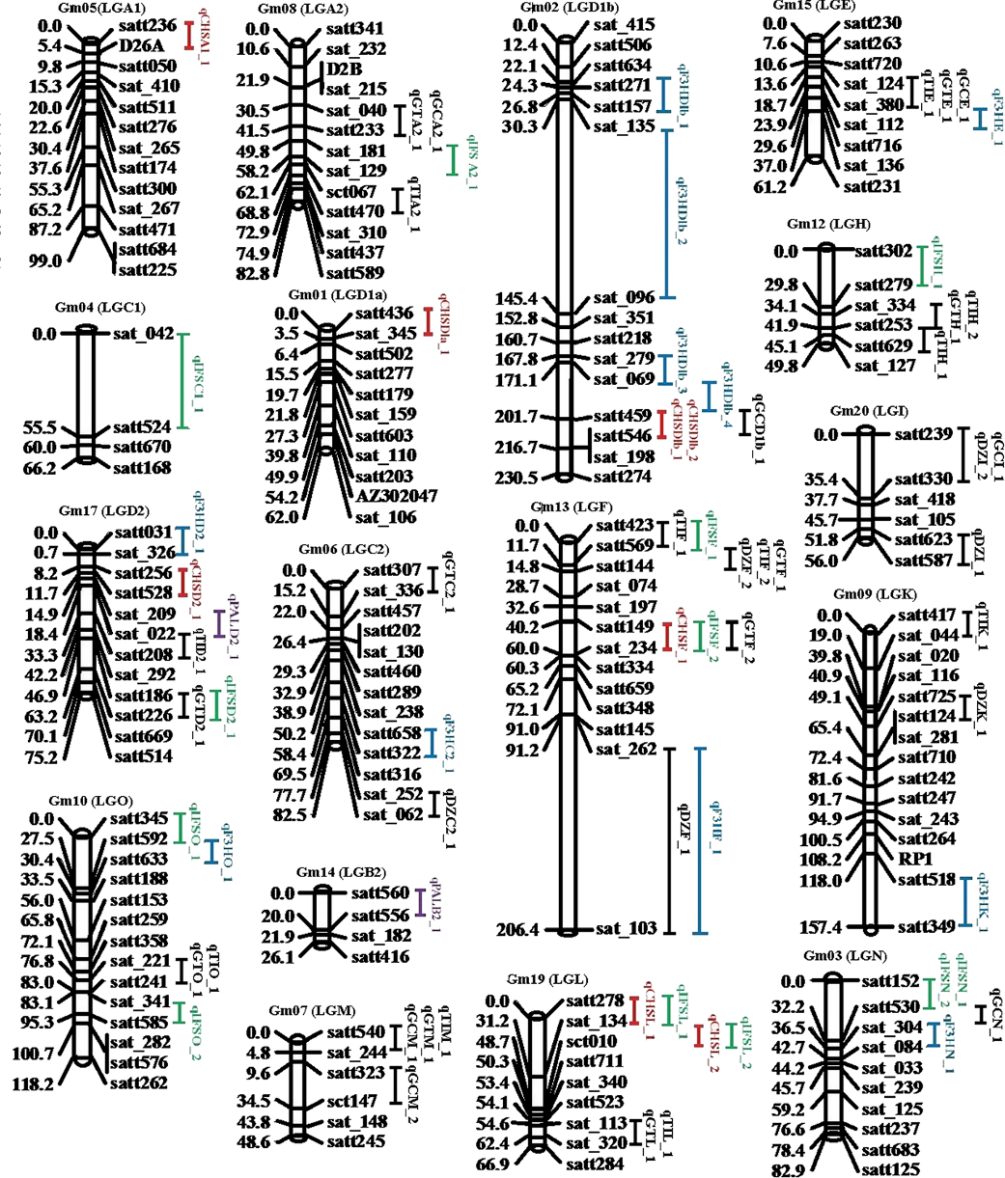
**Summary of eQTL and QTL locations detected in the soybean genome.** eQTL/ QTL represented by bars were shown on the left of the linkage groups, close to their corresponding markers. The lengths of the bars were proportional to the confidence intervals of the corresponding eQTL/QTL in which the inner line indicates the position of maximum LOD score.

The linkage map that included 194 SSR markers (accepted by Molecular Biology Reports) and covered 2,312 cM with mean distance of about 12 cM between markers was used to identify eQTLs associated with the expression of the four TGs. Thirty-three eQTLs that appeared to underlie transcript abundance of the four TGs are detected and located on fifteen LGs (Table 3, Figure 1). Regarding to the locational relationships between the eQTL and the genes, thirteen of the eQTLs were cis-acting (within 5 Mb upstream or downstream of the genes) and twenty of the eQTLs were trans-acting (more than 5 Mb away or on different chromosomes) [40, 53].

**Table 3:** **The eQTLs for target genes of** ***PAL, CHS*** **,** ***IFS*** **and** ***F3H***

Traits	eQTL^a^	Gm(LG)	Marker	Marker interval	Position^b^	Environment	LOD score	R^2^(%)^c^
*PAL*	^d^qPALB2_1	14(B2)	Satt560	Satt560 ~ Satt556	0.01	2011Harbin	3.39	8.11
	^d^qPALD2_1	17(D2)	Sat_209	Sat_209 ~ Sat_022	15.90	2011Harbin	4.24	6.67
*CHS*	qCHSA1_1	05(A1)	Satt 236	Satt 236-D26A	0.01	2011Harbin	5.48	4.21
	qCHSDla_1	01(Dla)	Satt436	Satt436-Sat_345	0.01	2011Harbin	8.64	2.71
	qCHSDlb_1	02(Dlb)	Satt546	Satt546-Satt459	214.80	2011Harbin	2.55	2.13
	qCHSDlb_2	02(Dlb)	Satt546	Satt546-Satt459	211.22	2011Harbin	2.18	3.90
	^d^qCHSD2_1	17(D2)	Satt528	Satt528-Satt256	10.74	2011Harbin	2.73	2.07
	qCHSF_1	13(F)	Sat_234	Sat_234-Satt149	46.17	2011Harbin	2.72	3.57
	qCHSL_1	19(L)	Satt278	Satt278-Sat_134	14.00	2011Harbin	2.40	15.65
	qCHSL_2	19(L)	Sat_134	Sat_134-Sct_010	24.51	2011Harbin	2.09	9.98
*IFS*	^d^qIFSA2_1	08(A2)	Sat_129	Sat_129-Sat_181	55.45	2011Harbin	7.46	17.67
	^d^qIFSC1_1	04(C1)	Sat_042	Sat_042-Satt524	6.67	2011Harbin	5.63	22.8
	^d^qIFSD2_1	17(D2)	Satt186	Satt186-Satt226	54.88	2011Harbin	8.87	16.67
	qIFSF_1	13(F)	Satt569	Satt569-Satt423	6.97	2011Harbin	3.09	15.84
	qIFSF_2	13(F)	Sat_234	Sat_234-Satt149	56.01	2011Harbin	10.92	17.89
	^d^qIFSH_1	12(H)	Satt302	Satt302-Satt279	0.01	2011Harbin	3.23	7.27
	^d^qIFSL_1	19(L)	Sat_134	Sat_134-Satt278	20.99	2011Harbin	7.26	16.12
	^d^qIFSL_2	19(L)	Sct_010	Sct_010-Sat_134	43.95	2011Harbin	9.75	17.97
	qIFSN_1	03(N)	Satt152	Satt152-Satt530	6.67	2011Harbin	2.50	27.42
	qIFSN_2	03(N)	Satt530	Satt530-Satt152	29.53	2011Harbin	2.50	12.80
	^d^qIFSO_1	10(O)	Satt345	Satt345-Satt592	6.00	2011Harbin	9.43	19.43
	^d^qIFSO_2	10(O)	Sat_341	Sat_341-Satt585	88.39	2011Harbin	9.78	15.69
*F3H*	^d^qF3HC2_1	06(C2)	Satt322	Satt322-Satt658	57.64	2011Harbin	2.27	2.37
	qF3HDlb_1	02(Dlb)	Satt157	Satt157-Satt271	25.71	2011Harbin	3.62	10.01
	^d^qF3HDlb_2	02(Dlb)	Sat_135	Sat_135-Sat_096	30.28	2011Harbin	7.53	14.32
	qF3HDlb_3	02(Dlb)	Sat_069	Sat_069-Sat_279	168.62	2011Harbin	2.41	8.49
	^d^qF3HDlb_4	02(Dlb)	Satt459	Satt459-Sat_069	185.58	2011Harbin	2.18	5.54
	^d^qF3HD2_1	17(D2)	Satt031	Satt031-Sat_326	0.01	2011Harbin	2.67	1.05
	^d^qF3HE_1	15(E)	Sat_112	Sat_112-Sat_380	22.09	2011Harbin	2.10	4.85
	^d^qF3HF_1	13(F)	Sat_262	Sat_262-Sat_103	101.22	2011Harbin	2.63	2.24
	^d^qF3HK_1	09(K)	Satt349	Satt349-Satt518	141.56	2011Harbin	2.08	1.57
	^d^qF3HN_1	03(N)	Sat_084	Sat_084-Sat_304	41.45	2011Harbin	4.70	6.10
	^d^qF3HO_1	10(O)	Satt592	Satt592-Satt633	27.54	2011Harbin	2.62	2.32

^a^eQTL: The nomenclature of the eQTL included four parts: QTL, trait, linkage group name and QTL order in the linkage group, respectively.

^b^Position from the left marker of the interval on each linkage group.

^c^Proportion of phenotypic variance (R^2^) explained by a eQTL.

^d^Trans-eQTL, others are cis-eQTL.

Among the identified eQTLs (Table 3), qPALB2_1 and qPALD2_1 were associated with *PAL* transcript abundance, and could explain 8.11% and 6.67% of the phenotypic variation, respectively. Eight eQTLs, underlying *CHS* transcript abundance, were located on six LGs, and could explain 2.07-15.65% of the phenotypic variation. qCHSDla_1 (Satt436-Sat_345, Gm01) was detected with a higher LOD score (8.64) in the regions where cis-elements and *CHS* family genes were located.

Two eQTLs (qCHSDlb_1, qCHSDlb_2), located in the interval of Satt459 and Satt546, could explain 2.13% and 3.90% of phenotypic variance and overlap with qGCD1b_1. qCHSF_1 (Satt149-Sat_234), associated with *CHS* and *IFS* transcript abundance, were overlapped with the marker interval of qGTF_2, and could explain 3.57% of phenotypic variance. qCHSL_1 (Satt278-Sat_134) and qCHSL_2 (Sat_134-Sct_010) were associated with the same SSR marker (Sat_134), and contributed 16.12% and 17.97% of the variation of *IFS* transcript abundance.

Twelve eQTLs were associated with *IFS* expression. Of them, qIFSD2_1 (Satt186-Satt226) explained 16.67% of the phenotypic variation. qIFSF_1 (Satt423-Satt569, R^2^ = 15.84%) shared the same SSR marker Satt569 with other three QTLs (qDZF_2, qGTF_1, qTIF_2). qIFSN shared the same SSR marker (Satt530) with qGCN_1 (Table 3, Figure 1).

Eleven eQTLs were associated with *F3H* expression (Table 3, Figure 1). Of them, four eQTLs were located on Gm02 (LG Dlb), and explained 5.54-14.32% of the phenotypic variation. qF3HDlb_2 (Sat_135-Sat_096) had higher LOD score and explained 14.32% of the phenotypic variation. qF3HE_1 (R^2^ = 4.85%) had the same interval (Sat_112- Sat_380) with qGCE_1, qGTE_1 and qTIE_1, meanwhile, qF3HF_1 and qDZF_1 shared the same marker interval (Sat_262- Sat_103) (Figure 1).

### Identification of candidate genes underlying the overlapped loci of pQTL and eQTL

Thirty four pQTLs for both individual and total seed isoflavone contents of soybean were compared with eQTLs to identify the overlapped loci. Five eQTL intervals were overlapped with pQTLs, and a total of eleven candidate genes within the overlapped eQTL and pQTL were identified (Table 4). Two genes, *C4H* (*Glyma02g40290.1*) and *PAL1* (*Glyma02g47940.1*), were identified on Gm02 (LG D1b) between Satt546-Satt459. *CHI* (*Glyma17g34430.1*) and *DFR* (dihydroflavonol reductase; EC 1.1.1.219) were identified on Gm17 (LG D2) between Satt186-Satt226. Genes encoding 4-coumarate-CoA ligase (EC 6.2.1.12; *Glyma13g01080.1/2*), *FLS* (*Glyma13g02740.1*) and *CHS* (*Glyma13g09640.1*) were identified on Gm13 (LG F) between Satt423-Satt569. Additionally, *CHS* (*Glyma13g24200.1*) and *IFS* (*Glyma13g09640.1*) was found within another eQTL/pQTL interval (Satt149-Sat_234).

**Table 4:** **Identification of candidate genes underlying overlapped locus of eQTL and QTL**

Marker interval	Gm(LG)	Physical location of markers	Candidate genes	Physical location of candidate genes	Function of candidate genes
Satt546-Satt459	Gm02(LGD1b)	43,775,407-48,390,089	*Glyma02g40290.1*	45,490,798-45,495,043	*C4H*
			*Glyma02g47940.1*	51,366,326-51,368,943	*PAL1*
Satt186-Satt226	Gm17(LG D2)	26,768,866-39,047,375	*Glyma17g34430.1*	38,398,978-38,401,025	*CHI*
			*Glyma17g37060.1*	40,920,379-40,923,898	*DFR*
Satt423-Satt569	Gm13(LG F)	5,231,035-9,567,285	*Glyma13g01080.1/2*	798,836-805,844	*4CL*
			*Glyma13g02740.1*	2,707,784-2,712,790	*FLS*
			*Glyma13g09640.1*	11,153,569-11,158,812	*CHS*
Satt149-Sat_234	Gm13(LG F)	4,976,740-26,460,745	*Glyma13g24200.1*	27,567,360-27,569,061	*IFS*
			*Glyma13g20800.1*	24,273,025-24,278,037	*PAL1*
			*Glyma13g27380.1*	30,577,113-30,579,230	*DFR*
			*Glyma13g09640.1*	11,153,569-11,158,812	*CHS*
			*Glyma13g02740.1*	2,707,784-2,712,790	*FLS*
Sat_262-Sat_103	Gm13(LG F)	7,233,012-25,478,474	*Glyma13g20800.1*	24,273,025-24,278,037	*PAL1*
			*Glyma13g24200.1*	27,567,360-27,569,061	*IFS*
			*Glyma13g09640.1*	11,153,569-11,158,812	*CHS*
			*Glyma13g02740.1*	2,707,784-2,712,790	*FLS*

## Discussion

Soybean isoflavones have been broadly used in food, medicine, cosmetics and animal husbandry [59]. Increasing and decreasing seed isoflavone content will be an important target of soybean breeding. MAS based on genotype selection rather than solely on phenotype selection provides additional power for the selections during soybean breeding [60]. Cultivar ‘Zhongdou 27’ proved to have high-isoflavone content (3,791 μg/g isoflavone in seed) as reported previously [16]. Meng et al. [19] identified two QTL underlying resistance to soybean aphid through leaf isoflavone-mediated antibiosis in soybean cultivar ‘Zhongdou 27’. A number of pQTLs associated with seed isoflavone were identified in multiple environments from cultivar ‘Zhongdou 27’ using 194 SSR markers (accepted by Molecular Biology Reports). Therefore, ‘Zhongdou 27’ should be given more attention as an elite germplasm to improve soybean seed isoflavone concentration, disease and pest resistances.

In our previous studies, some identified QTLs associated with individual/total isoflavone contents showed higher contribution to phenotypic variation. Some specific copies of genes (*PAL*, *CHS*, *IFS*, *F3H*) in the phenylpropanoid pathway were near or falling into these quantitative trait loci by browsing the reference genome sequence of Williams 82 (http://www.phytozome.net/soybean).

To investigate the regulation mechanism of isoflavone synthetic enzyme genes, the transcript abundances of *PAL*, *CHS*, *IFS* and *F3H* in the mapping population were examined, and the genomic regions affecting the expression of the TGs were identified using the eQTL methodology [61]. A global microarray eQTL analysis of a limited number of samples can be used for exploring functional and regulatory gene networks and for scanning cis-eQTL, whereas the subsequent analysis of a subset of likely cis-regulated genes by real-time RT-PCR in a larger number of samples may identify QTL region by targeting these positional candidate genes [62]. In this study, real-time PCR reactions were used to analyze the transcript abundance variations of the four TGs in the F_5:11_ RI lines.

When combined with classical QTL phenotypes, correlation analysis can directly provide an overview of potential genes underlying isoflavone traits [63, 64]. Through the comparison of the transcript abundances of the four TGs (*PAL*, *CHS*, *IFS* and *F3H*), the parents (‘Zhongdou 27’ and ‘Jiunong 20’) showed different patterns at the R6 stage. This observation was consistent with the previous report by Sarah et al. [65]. Significant correlations between the transcript abundances of TGs and isoflavone contents were found in developing seeds at the R6 stage, indicating that these genes could affect total and individual isoflavone accumulations (Table 2).

Previously, two major QTLs that affect isoflavone content across multiple environments were mapped on Gm05 (LG A1) and Gm08 (LG A2) by Gutierrez et al. [17] and Yang et al. [20], respectively. In the present work, one eQTL qIFSA2_1 (Sat_129-Sat_181) was mapped close to qGCA2_1 on Gm08 (LG A2) (Figure 1, Table 5). This result suggested that qIFSA2_1 might be a cis-enzyme related locus. Some of these identified eQTLs associated with seed isoflavone content did not coincide with the TGs, suggesting that the differences in TGs transcript abundances might be caused by several trans-acting factors [66].

**Table 5:** **Partial QTLs for individual and total isoflavone contents**

Traits^a^	QTL^b^	Gm (LG)	Marker	Marker interval	Position^c^	Environment^d^	LOD score	R^2^(%)^e^
DZ	^f^qDZF_1	13(F)	Sat_103	Sat_103-Sat_262	188.34	E2	2.00	10.57
GC	qGCA2_1	08(A2)	Sat_040	Sat_040-Satt233	38.46	E3	2.65	6.01
	^f^qGCD1b_1	02(Dlb)	Satt546	Satt546-Sat_459	215.67	E2	2.38	3.12
						E5	2.21	4.17
GT	^f^qGTD2_1	17(D2)	Satt186	Satt186-Satt226	50.81	E1	2.00	3.41
						E2	2.36	5.23
						E3	5.76	10.98
						E5	3.09	8.23
	^f^qGTF_2	13(F)	Satt149	Satt149-Sat_234	41.23	E1	2.00	1.56
						E3	2.49	4.17
						E7	4.03	5.47
TI	^f^qTIF_1	13(F)	Satt423	Satt423-Satt569	6.01	E6	4.59	3.21
						E7	2.15	4.2

^a^DZ: Daidzein; GC:Glycitein; GT: Genistein; TI: Total isoflavone.

^b^The nomenclature of the QTL included four parts : QTL, trait, linkage group name and QTL order in the linkage group, respectively.

^c^Position from the left marker of the interval on each linkage group.

^d^E1: at Harbin in 2005, E2: at Harbin in 2006, E3: at Hulan in 2006, E4:at Suihua in 2006, E5: at Harbin in 2007, E6: at Hulan in 2007, E7: at Suihua in 2007.

^e^Proportion of phenotypic variance (R2) explained by a QTL.

^f^Overlapped loci of pQTL and eQTL.

In this study, since the 194 markers were not uniformly distributed, large gaps appeared with low marker density on chromosomes Gm02, 04, 13, 16 and 18, implying that more markers should be developed among these gaps and the authenticity of pQTL or eQTL should be further clarified. Among these gaps, special attention should be paid to eQTL qF3HDlb_2 on chromosome Gm02 and qIFSC1_1 on chromosome Gm04 because of their higher LOD score and contribution to phenotypic variation (Table 3). Overlapped loci of qF3HF_1 and qDZF_1, and genes that fall into this region should also be further clarified with more markers. Consequently, fine mapping on these intervals with more SSR or SNP markers and to determine the authenticity of these loci as well as the underlying genes were extremely essential in the future work.

The analysis of eQTL overlapped with pQTL suggested that the candidate genes or elements among the marker intervals could affect phenotypic traits [49, 67, 68]. Therefore, overlapped loci of eQTLs and pQTLs were analyzed to find the potential candidate genes affecting the accumulation of isoflavone contents in soybean seed. Five eQTL intervals were overlapped with pQTLs according to the comparison of genomic regions between pQTLs and eQTLs (Table 5). These results indicated that some candidate genes or elements in these intervals could regulate the biosynthesis of isoflavone components, and affect their accumulation. Additionally, some eQTLs overlapped with other eQTLs or shared the same markers with pQTLs, suggesting that some candidate genes or elements were located near these loci.

Several genes involved in isoflavone accumulation in soybean seed had been identified [22, 27, 31]. 11 candidate genes falling into the overlapped intervals of pQTL and eQTL were found (Table 4). Bolon et al. [58] identified eQTL for genes with seed-specific expression and discovered striking eQTL hotspots at distinct genomic intervals on chromosome Gm13. A chalcone isomerase (*CHI3*) and *IFS2* gene were located in the same region identified by qGEN13 on Gm13 [11]. Another QTL for GC that encoded *PAL* and *4CL* paralog was also reported on Gm13 [10, 11]. In the present work, seven candidate genes on Gm13 (LG F) were identified, implying that there could be a hotspot of gene cluster that regulated seed isoflavone content on Gm13. Among them, *CHS* (*Glyma13g09640.1*) and *FLS* (*Glyma13g02740.1*) were identified on three overlapped loci, implying that they could interact or trans-regulate other genes in the phenylpropanoid pathway. Furthermore, *PAL1* (*Glyma13g20800.1*) and *IFS* (*Glyma13g24200.1*) paralogs were identified within two overlapped loci. In the marker interval (Satt149-Sat_234) associated with qCHSF_1, qIFSF_2 and qGTF_2, both *Glyma13g24200.1* and *Glyma13g09640.1* were found to encode *CHS* and *IFS*, indicating that they could be the potential candidate genes. It was supposed that *Glyma13g09640.1* could interact or trans-regulate the expression of *IFS*. However, the function of these potential candidate genes should be tested in future works.

Although open questions about the biology and applications of eQTL mapping still exist [69], there are considerable advances in the eQTL studies. Detailed analysis of eQTL combined with cluster analysis of transcript abundance and eventually gene expression patterns could assist map-based cloning of genes underlying these traits. Markers based on underlying genes are also desirable for MAS in soybean breeding programs. The mechanism underlying seed isoflavone synthesis and its accumulation may contribute to the development of marker-assisted selection for soybean cultivars with high or low isoflavone contents.

## Conclusions

A total of thirty three eQTLs (thirteen cis-eQTLs and twenty trans-eQTLs) were identified on fifteen chromosomes. Five eQTL intervals were overlapped with pQTLs and a total of eleven candidate genes within the overlapped eQTL and pQTL were identified. These results might be beneficial for the development of marker-assisted selection to breed soybean cultivars with high isoflavone contents.

## Methods

### Plant materials and growing conditions

The mapping population of 130 F_5:11_ recombinant inbred (RI) lines were derived through single-seed-descent from the cross between ‘Zhongdou 27’ (developed by the Chinese Academy of Agricultural Sciences, Beijing, China) and ‘Jiunong 20’ (developed by Jilin Academy of Agricultural Sciences, Jilin, China). ‘Zhongdou 27’ contains high individual and total isoflavone (TI) contents in seed (daidzein, DZ, 1,865 μg/g; genistein, GT, 1,614 μg/g; glycitein, GC, 311 μg/g and total isoflavone, TI, 3,791 μg/g), whereas ‘Jiunong 20’ has low individual and TI contents (DZ, 844 μg/g; GT, 1,046 μg/g; GC, 193 μg/g and TI, 2,061 μg/g).

To detect eQTL, the parents and the 130 F_5:11_ RI lines were planted at Harbin, Heilongjiang Province, China, in 2011. Randomized complete block designs were used for all experiments with rows 3 m long, 0.65 m apart, and a space of 6 cm between plants. Mature and immature seeds in the reproductive stages (from soybean growth stage R3 to R8) [70] were harvested from a bulked sample collected from three plants in each plot. These samples were quantified for individual and total seed isoflavone contents and transcript abundances.

### Isoflavone extraction and quantification

Approximately 150 g of soybean seed samples were ground to a fine power using a commercial coffee grinder. Isoflavones were extracted from flour and separated using HPLC as described previously [16]. Measurements were done as micrograms of isoflavone per gram of seeds plus and minus the standard deviations (μg/g ± SD).

### Synthesis of cDNA, Real-Time PCR and data collection

To investigate the expressions of four TGs, total RNA was isolated from soybean seed samples from R3 to R8 stages using plant RNA purification reagent Kit (D9108A, TaKaRa, Japan). RNAs were transcribed to cDNA using the first strand DNA synthesis reagent Kit (D6110A, TaKaRa, Otsu, Shiga, Japan). Four TGs (*PAL*, GenBank accession: GQ220305; *CHS*, GenBank accession: EU526827; *IFS*, GenBank accession: FJ770473 and *F3H*, GenBank accession: AY595420) in the phenylpropanoid pathway, were selected to analyze the transcript abundance variations in the F_5:11_ RI line population. These four TGs were analyzed by real-time PCR (Kit DRR081A, TaKaRa, Japan). Gene-specific primers for expression analysis of the four TGs were listed in Table 6. Primer specificity was confirmed based on each primer pair sequence against soybean genome sequences by BLASTing (http://www.phytozome.net/soybean) using the BLASTN algorithm. Moreover, through the BLASTN of the sequences of the TGs, *PAL2* (located on Gm10 (LG O)) of the *PAL* gene family, *CHS8* (located on Gm11 (LG B1)) of the *CHS* gene family, *IFS1* (located on Gm07 (LG M)) of the *IFS* gene family, and *F3H1* and *F3H2* (located on Gm02 (LG D1b)) of the *F3H* gene family were amplified [11].

**Table 6:** **Real-time PCR primer pairs for the expression analyses of** ***PAL*** **,** ***CHS*** **,** ***F3H*** **, and** ***IFS*** **genes**

Gene	Forward primer (5′-3′)	Reverse primer (5′-3′)	PCR product length (bp)
*Actin4*	GTGTCAGCCATACTGTCCCCATTT	GTTTCAAGCTCTTGCTCGTAATCA	214
*PAL*	ATTATGGATTCAAGGGAGCT	AATGAGGAAAGTGGAGGACA	182
*CHS*	AAAATGCCATCTCCTCAAACA	GGATCTCAGCTACGCTCACC	155
*F3H*	GCTTGCGAGAATTGGGGTAT	CCTTGGAGATGGCTGGAGAC	176
*IFS*	GCCCTGGAGTCAATCTGG	CAAGACTATGTGCCCTTGGA	171

PCR amplification was performed as follows: 95°C for 60 s, followed by 40 cycles of 95°C for 11 s, 60°C for 12 s and 72°C for 18 s. The soybean *actin4* (GenBank accession: AF049106) gene was used as a reference to quantify the expression levels of the target genes [71]. Three replicates for each reaction were performed. The relative transcript abundance of TGs in different samples was calculated using 2^-ΔΔCt^ method [72], defined as: ΔCt = Ct (target) – Ct (actin). Pearson correlations between total/individual isoflavone contents and the expression of the four TGs in F_5:11_ RILs were evaluated using SAS 8.2 (Cary, NC, USA) [73].

### Identification of genomic region of target genes

The whole genome sequence Glyma1 assembly for Williams 82 [74] provided a powerful tool for interrogating QTL data. Previously reported genes for isoflavone biosynthesis [75] were used in BLAST searches against the whole genome sequence to identify homologous regions in the genome with assigned or putative functions. All twenty soybean chromosomes have regions sharing a high percentage of homology with genes of known function in the phenylpropanoid pathway [11]. The coding regions of TGs were compared with genome of Williams 82 through BLAST (E-value ≤ 1.0E-05, http://www.phytozome.net/soybean) to identify homologous regions.

### eQTL analysis

In previous work, fifteen QTL underlying seed isoflavone contents of soybean were identified based on RI line populations derived from a cross between ‘Zhongdou 27’ (high isoflavone) and ‘Jiunong 20’ (low isoflavone) through a genetic linkage map including 99 SSR markers [16]. Another 95 SSR markers were added to the map of Zeng et al. [16] to identify novel phenotypic QTLs (pQTLs) associated with seed isoflavone contents of soybean (accepted by Molecular Biology Reports). In this study, 194 polymorphic markers were assembled onto the 20 linkage groups (LGs) by Mapmaker 3.0b with the Kosambi mapping function [76]. WinQTLCart2.1 [77] was used to detect eQTL between marker intervals by 1,000 permutations at significance (*P ≤ 0.05*). The genetic linkage map was constructed using Mapchart 2.1 [78]. The nomenclature of the eQTLs/pQTLs included four parts following the recommendations of the Soybean Germplasm Coordination Committee. For example, qCHSF_1, q, CHS, F and 1 represent eQTL, trait (*CHS*), linkage group name and eQTL order in the linkage group, respectively.

### Identification of candidate genes underlying overlapped loci of pQTL and eQTL

Coincident genetic locations of eQTL and pQTL may be available to identify important regulatory genes underlying traits, and lead to the identification of molecular mechanisms [49, 67, 68]. Previous studies have combined eQTL and pQTL mapping to gain insight into regulatory pathways involved in determining phenotypic traits [49, 68, 79–81]. eQTL located in the same marker intervals of pQTL might contribute to significant phenotypic variations [49, 67, 68]. In this study, thirty four phenotypic QTL (pQTL) identified with the 194 SSR markers were compared with eQTL to identify overlapped loci. Genetic map positions were estimated by identifying the nearest flanking SSR markers using the genome browser (http://www.soybase.org). The candidate genes underlying overlapped loci of pQTL and eQTL were identified by browsing after using BLAST search of flanking markers against the whole genome sequence of Williams 82 (available at: http://www.phytozome.net/soybean).
